# Comparative Effectiveness of Coronary Artery Bypass Graft Surgery and Percutaneous Coronary Intervention for Patients With Coronary Artery Disease: A Meta-Analysis of Randomized Clinical Trials

**DOI:** 10.7759/cureus.29505

**Published:** 2022-09-23

**Authors:** Tanveer Ahamad Shaik, Sandipkumar S Chaudhari, Taimur Haider, Ramza Rukia, Saman Al Barznji, Heemali Kataria, Laxman Nepal, Adil Amin

**Affiliations:** 1 Cardiovascular Medicine, University of Louisville School of Medicine, Louisville, USA; 2 General Practice, Lions General Hospital, Mehsana, IND; 3 General Practice, General Hospital, Vadnagar, IND; 4 Internal Medicine, District Headquarter Hospital, Lahore, PAK; 5 Medical Student, Liaquat University of Medical and Health Sciences, Hyderabad, PAK; 6 Internal Medicine, University of Sulaymaniyah, Sulaymaniyah, IRQ; 7 Internal Medicine, Government Medical College, Surat, Surat, IND; 8 Internal Medicine, Kathmandu Medical College and Teaching Hospital, Kathmandu, NPL; 9 Cardiology, Pakistan Navy Station Shifa, Karachi, PAK

**Keywords:** efficacy, meta-analysis, coronary artery disease, coronary artery bypass graft surgery, percutaneous coronary intervention

## Abstract

Percutaneous coronary intervention (PCI) and coronary artery bypass graft (CABG) surgery are the options for revascularization in coronary artery disease (CAD). This meta-analysis aims to compare the efficacy of CABG and PCI for the management of patients with CAD. The meta-analysis was conducted as per the Preferred Reporting Items for Systematic Reviews and Meta-Analyses (PRISMA) guidelines. PubMed, Cochrane Library, and EMBASE were searched for relevant articles. The reference list of included articles was also searched manually for additional publications. Primary endpoints were cardiovascular mortality and all-cause mortality. Secondary endpoints included myocardial infarction, stroke, and revascularization. In total, 12 randomized control trials (RCTs) were included in this meta-analysis encompassing 9,941 patients (4,954 treated with CABG and 4,987 with PCI). The analysis showed that PCI was associated with a higher risk of all-cause mortality (risk ratio (RR) = 1.26, 95% confidence interval (CI) = 1.10-1.45) and revascularization (RR = 2.42, 95% CI = 1.82-3.21). However, no significant differences were reported between two arms regarding cardiovascular mortality (RR = 1.15, 95% CI = 0.96-1.39), myocardial infarction (RR = 1.17, 95% CI = 0.82-1.67), and stroke (RR = 0.64, 95% CI = 0.35-1.16). CABG was associated with a significant reduction in all-cause mortality and revascularization compared to PCI. However, no significant difference was reported in the risk of cardiovascular mortality, myocardial infarction, and stroke between the two groups.

## Introduction and background

Coronary artery disease (CAD) is a major cause of mortality worldwide [1]. The complexity and severity of CAD can vary among patients. CAD can involve a single vessel and can impact various territories such as multivessel coronary disease. CAD can also impact arteries with little to no clinical significance or arteries vital to the survival and function of the left ventricle, including the left main coronary artery. For the past several years, coronary artery bypass grafting (CABG) has been the standard of care for invasive treatment of left main and multivessel CAD, considering its extensive advantage in survival [2]. However, in the last few decades, rapid advancements have been made in percutaneous coronary intervention (PCI), including pharmacotherapy, adjunctive imaging support, and stent technology [2]. These have enhanced the surgical approach to the treatment of CAD. Based on the results of small randomized control trials (RCTs) [3,4], with the above-mentioned technical and pharmacological advancements, the value of PCI in the treatment of CAD is still being explored. RCTs including NOBLE [3] and Excel [4] trials have added some uncertainty to this vital topic.

Currently, and with large numbers of RCTs being performed among patients with multivessel and left main artery CAD, the choice of suitable coronary artery revascularization strategy remains unclear [5]. RCTs by Head et al. (2014) and Farkouh et al. (2012) [6,7], along with large retrospective studies [8,9], have all reported consistent findings preferring CABG over PCI for long-term benefits. The NOBLE trial showed that in individuals with left main CAD [3], PCI was less effective than CABG, while the Excel trial showed non-inferiority of PCI compared to CABG [4]. The results of these trials are different because of their different methodologies, and, therefore, their results need to be interpreted with caution.

A suboptimal outcome was obtained following PCI in individuals with a high-risk profile who were ruled inoperable for CABG. Patients who cannot undergo PCI because of the complexity of CAD benefit greatly from bypass surgery [10]. The study by Kappetein et al. found that patients with a complex disease have a greater risk for major adverse cardiovascular events and all-cause mortality with PCI, making CABG the preferred treatment option [11]. In left main CAD, CABG can significantly reduce major cardiac-related events compared to PCI [12].

The recommendations for PCI are somewhat weaker despite the fact that more recent data indicate that PCI may sometimes produce results that are comparable to, if not better than, CABG [3]. Nowadays, most patients prefer a less invasive approach. Moreover, robust data are important for the facilitation of appropriate choices for individual patients. Since the current recommendations were issued, numerous clinical trials comparing PCI and CABG in various patient subgroups have been performed. Therefore, it is essential to conduct a current meta-analysis that takes this data into account. This meta-analysis aims to compare the efficacy of CABG and PCI for the management of patients with CAD. This meta-analysis analyzed the complete spectrum of stable and unstable coronary syndromes across a gamut of different subgroups of patients.

## Review

Methodology

This meta-analysis was conducted as per the Preferred Reporting Items for Systematic Reviews and Meta-Analyses (PRISMA) guidelines.

Search Strategy and Study Selection

Two reviewers independently searched electronic databases from inception to August 1, 2022, including PubMed, Cochrane Library, and EMBASE without putting restrictions on the year of publication and language. The reference lists of included articles were also searched manually for additional publications. Keywords used to search for relevant articles were “coronary artery bypass graft,” “percutaneous coronary intervention,” and “coronary artery disease.” This meta-analysis includes RCTs that compared PCI and CABG for the management of CAD in the presence of left main CAD, multivessel CAD, or both. Observational studies, cross-over trials, and reviews were excluded from this meta-analysis. Second, we excluded studies that compared CABG or PCI along with medical therapy and excluded studies that compared two forms of CABG and that compared two forms of PCI.

Two authors reviewed the titles and abstracts of the articles independently, followed by full-text screening, as required for determining whether the studies fulfilled the eligibility criteria. Conflicts between authors were resolved through discussion and re-review.

Outcome Measures

The primary endpoints were cardiovascular mortality and all-cause mortality. Secondary endpoints included myocardial infarction, stroke, and repeat revascularization. Only studies with a minimum follow-up of one year were included.

Quality Assessment

The risk of bias assessment of each included study was done by two authors independently using the criteria defined in the Cochrane Handbook for Systematic Reviews of Interventions. The risk of bias was assessed in the following six domains: random sequence generation, allocation concealment, blinding of participants and personnel, blinding of outcome assessment, incomplete outcome data, selective outcome reporting, and other biases. Each domain was graded as high, low, or unclear for each of the included studies. Conflicts between authors were resolved through discussion and re-review.

Data Extraction

Two review authors extracted study characteristics from included studies using pre-designed data collection forms. The following data were extracted from each of the included studies: the first author, year of publication, sample size, follow-up duration, patient gender, patient age, percentage of patients with diabetes, hypertension, baseline SYNTAX score, and outcomes. Conflicts between authors were resolved through discussion and re-review. One author transferred the data into the Review Manager File for analysis, and one author double-checked whether the data was put correctly by comparing it with the completed data collection form.

Statistical Analysis

Dichotomous data were presented as risk ratio (RR) with 95% confidence intervals (95% CI) using the Mantel-Haenszel model. The extent of heterogeneity was assessed using the I^2^ statistics and Cochran Q test. I^2^ values of 0-25%, 25-50%, and 75-100% denote low, moderate, and high heterogeneity, respectively. If there was evidence for homogenous effects across trials (I^2^ <50%), we used RR to analyze the data and the fixed-effects model to summarize all results. If we discovered significant levels of heterogeneity, as shown by a high I2 statistic value of at least 50%, we used the random-effects model. Publication bias for each of the outcomes was assessed using Egger’s test. Stratified analyses were done for early-generation drug-eluting stents (DES) and bare-metal stents (BMS) or newer-generation DES, and for left main CAD and multivessel CAD. Early generation DES included sirolimus-eluting and paclitaxel-eluting stents, while newer generation DES included everolimus-eluting and zotarolimus-eluting stents. For a subgroup analysis of studies, data for primary outcomes (all-cause mortality and cardiac-related death) were extracted to calculate RR. Analysis was performed using Review Manager version 5.4.1 (Cochrane, London, UK) and STATA version 16.0 (STATA Corporation, College Station, TX, USA).

Results

**Figure 1:**
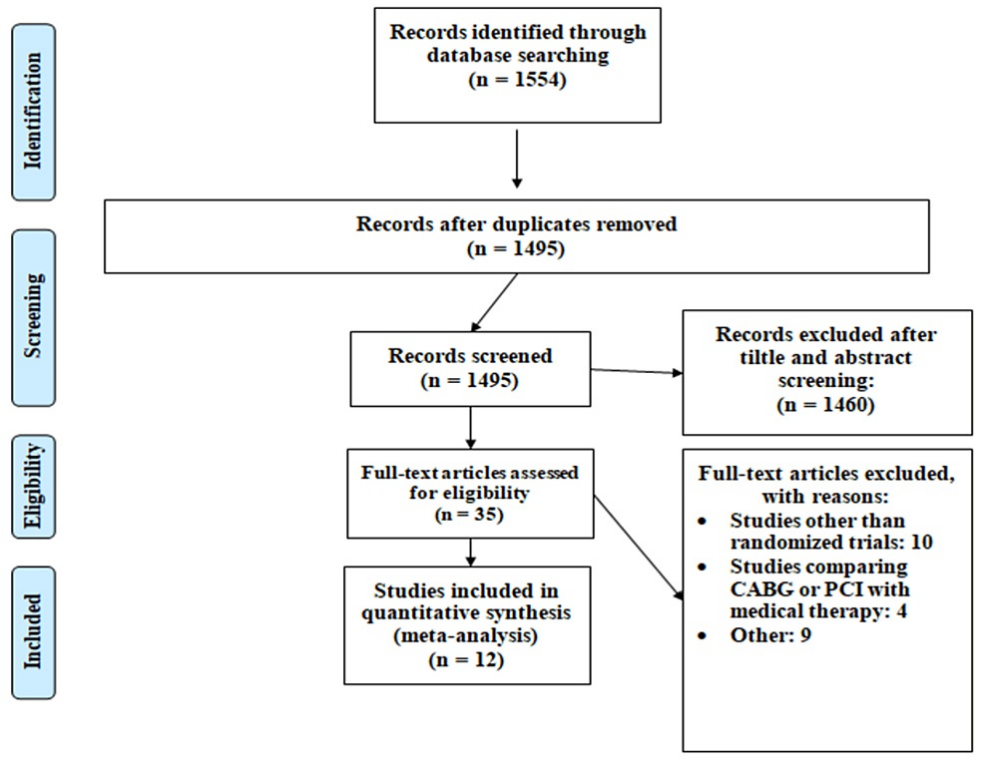
PRISMA flowchart of selection of studies. CABG: coronary artery bypass graft; PCI: percutaneous coronary intervention; PRISMA: Preferred Reporting Items for Systematic Reviews and Meta-Analyses

Figure 1 shows the PRISMA flowchart of the selection of studies. Out of a total of 1,554 articles resulting from the initial database literature search, 1,495 articles were retrieved for abstract and title analysis. Among 1,495 articles, the full text of 35 articles was accessed to assess eligibility. In total, 12 studies fulfilled the inclusion criteria and were included in this meta-analysis. These RCTs enrolled a total of 9,941 patients, of whom 4,954 were assigned to CABG and 4,987 were assigned to PCI. Table 1 shows the general characteristics of the included studies.

**Table 1:** Characteristics of the included studies. CAD: coronary artery disease; CABG: coronary artery bypass graft; PCI: percutaneous coronary intervention

Author	Year	Setting	Population	Groups	Sample size	Follow-up period
Boudriot et al. [13]	2011	Multicenter	Left main CAD with or without multivessel CAD	PCI	100	1 year
				CABG	101	
Booth et al. [14]	2008	Multicenter	Multivessel coronary disease	PCI	488	6 years
				CABG	500	
Buszman et al. [15]	2008	Single center	Left main CAD with or without multivessel CAD	PCI	52	1 year
				CABG	53	
Farkouh et al. [7]	2012	Multicenter	Multivessel coronary disease	PCI	953	5 years
				CABG	947	
Hueb et al. [16]	2009	Single center	Multivessel coronary disease	PCI	205	10 years
				CABG	203	
Kamalesh et al. [17]	2013	Multicenter	Multivessel coronary disease	PCI	101	2 years
				CABG	97	
Kapur et al. [18]	2010	Multicenter	Multivessel coronary disease	PCI	256	1 year
				CABG	248	
Kumar et al. [19]	2020	Single center	Multivessel coronary disease	PCI	103	1 year
				CABG	107	
Mäkikallio et al. [3]	2016	Multicenter	Patients with left main CAD	PCI	592	5 years
				CABG	592	
Park et al. [20]	2011	Multicenter	Left main CAD	PCI	300	2 years
				CABG	300	
Serruys et al. [21]	2009	Multicenter	Left main and/or three-vessel disease	PCI	891	1 year
				CABG	849	
Stone et al. [4]	2016	Multicenter	Patients with left main CAD	PCI	948	3 years
				CABG	957	

**Figure 2:**
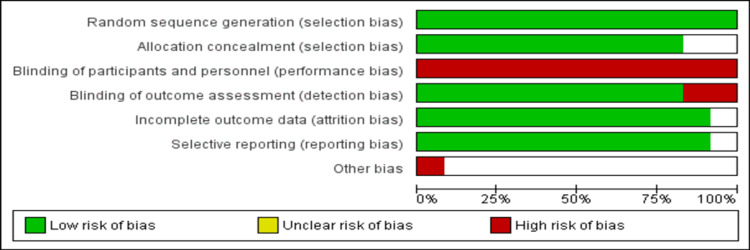
Graph showing the risk of bias.

Nine studies were multicenter [3,4,7,13,14,17,18,20,21]. The follow-up of included RCTs ranged from one year to ten years. Figure 2 represents the risk of bias of the included studies. Two reviewers assessed the risk of bias, and it was found to be consistent. The overall study quality was good.

Table 2 presents the characteristics of patients enrolled in RCTs included in this meta-analysis. The pooled mean age of patients was 62.88 years. Patients enrolled were mainly males (75.9%). More than one-third of the participants were diabetic (44.2%), and nearly two-thirds of patients had hypertension (65.14%).

**Table 2:** Characteristics of participants. *: Mean (standard deviation) UK: not given in the article; CABG: coronary artery bypass graft; PCI: percutaneous coronary intervention

Author	Groups	Age*	Male n (%)	Diabetes n (%)	Hypertension n(%)	Baseline SYNTAX score*
Boudriot et al., 2011 [13]	PCI	66 (8.1)	72 (72)	40 (40)	82 (82)	UK
	CABG	69 (7.4)	78 (77)	33 (33)	83 (82)	UK
Booth et al., 2008 [14]	PCI	61 (9.2)	390 (80)	68 (13.9)	212 (43)	UK
	CABG	62 (9.5)	392 (78)	74 (14.8)	235 (47)	UK
Buszman et al., 2008 [15]	PCI	60.6 (10.5)	31 (60)	10 (19)	39 (75)	25.2 (8.7)
	CABG	61.3 (8.4)	39 (73)	9 (17)	37 (70)	24.7 (6.8)
Farkouh et al., 2012 [7]	PCI	63.2 (8.9)	698 (73.2)	953 (100)	UK	26.2 (8.4)
	CABG	63.1 (9.2)	658 (69.5)	947 (100)	UK	26.1 (8.8)
Hueb et al., 2009 [16]	PCI	60 (9)	138 (67)	47 (23)	125 (61)	UK
	CABG	60 (9)	146 (72)	59 (29)	128 (63)	UK
Kamalesh et al., 2013 [17]	PCI	62.7 (7.1)	100 (99)	101 (100)	97 (96)	21.5 (8.9)
	CABG	62.1 (7.4)	96 (99)	97 (100)	90 (95.7)	22.7 (10.6)
Kapur et al., 2010 [18]	PCI	64.3 (8.5)	181 (70.7)	256 (100)	196 (76.6)	UK
	CABG	63.6 (9.1)	197 (77.9)	248 (100)	203 (80.6)	UK
Kumar et al., 2020 [19]	PCI	59 (9)	64 (62)	21 (20)	45 (44)	UK
	CABG	59 (10)	65 (61)	22 (21)	46 (43)	UK
Mäkikallio et al., 2016 [3]	PCI	66·2 (9·9)	476 (80)	86 (15)	386 (65)	22·5 (7·5)
	CABG	66·2 (9·4)	452 (76)	90 (15)	389 (66)	22·4 (8·0)
Park et al., 2011 [20]	PCI	61.8 (10)	228 (76)	102 (34)	163 (54.3)	UK
	CABG	62.7 (9)	231 (77)	90 (30)	154 (51.3)	UK
Serruys et al., 2009 [21]	PCI	65.2 (9.7)	681 (76.4)	228 (25.6)	UK	28.4 (11.5)
	CABG	65 (9.8)	670 (78.9)	209 (24.6)	UK	29.1 (11.4)
Stone et al., 2016 [4]	PCI	66 (9.6)	722 (76.2)	286 (30.2)	703 (74.2)	20.6 (6.2)
	CABG	65.9 (9.5)	742 (77.5)	268 (28.0)	701 (73.2)	20.5 (6.1)

All-Cause Mortality and Cardiovascular Mortality

**Figure 3:**
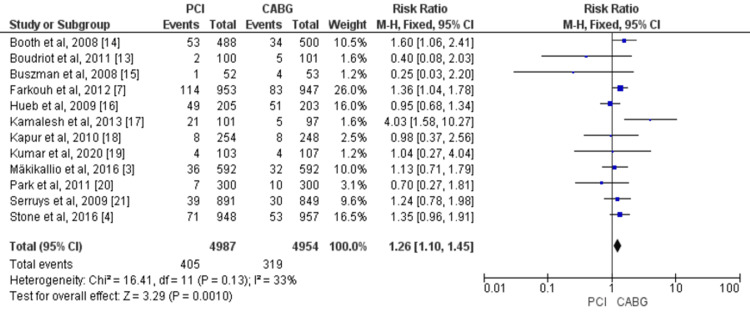
Pooled risk for all-cause mortality with PCI versus CABG. Sources: References [3,4,7,13-21]. CABG: coronary artery bypass graft; PCI: percutaneous coronary intervention; 95% CI: 95% confidence interval

Overall, 12 studies assessed all-cause mortality by enrolling 9,941 patients (4,954 treated with CABG and 4,987 with PCI) [3,4,7,13-21]. The pooled data of included studies revealed that the risk of all-cause mortality was significantly higher in patients treated with PCI compared to CABG (RR = 1.26, 95% CI = 1.10-1.45). No significant heterogeneity was found among the study results (p-value = 0.13, I^2^ = 33%), as shown in Figure 3.

**Figure 4:**
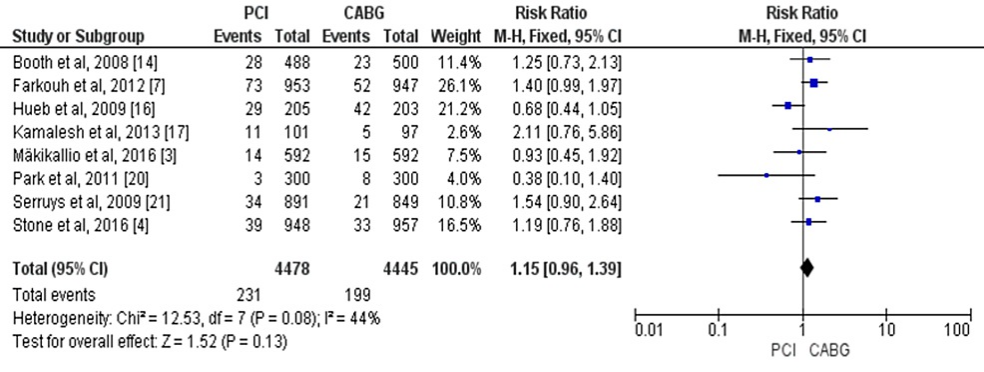
Pooled risk for cardiac-related mortality with PCI versus CABG. Sources: Reference [3,4,7,14,16,17,20,21]. CABG: coronary artery bypass graft; PCI: percutaneous coronary intervention; 95% CI: 95% confidence interval

No significant difference was found between CABG and PCI regarding cardiovascular mortality (eight studies, 8,923 patients; RR = 1.15, 95% CI = 0.96-1.39). Significant heterogeneity was found among the study results (p-value = 0.08, I^2^ = 44%), as shown in Figure 4.

Myocardial Infarction, Stroke, and Revascularization

**Figure 5:**
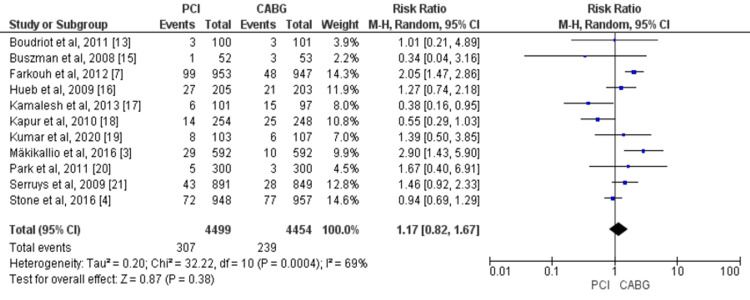
Pooled risk for myocardial infarction with PCI versus CABG. Source: References [3,4,7,13,15-21]. CABG: coronary artery bypass graft; PCI: percutaneous coronary intervention; 95% CI: 95% confidence interval

Overall, 11 studies compared the risk of myocardial infarction between two study groups including 8,953 patients with CAD (4,499 in the PCI group and 4,454 in the CABG group) [3,4,7,13,15-21]. Myocardial estimates from the random-effect model showed no significant difference in myocardial infarction between the PCI and CABG arm (RR = 1.17, 95% CI = 0.82-1.67, I^2^ = 69%), as shown in Figure 5.

**Figure 6:**
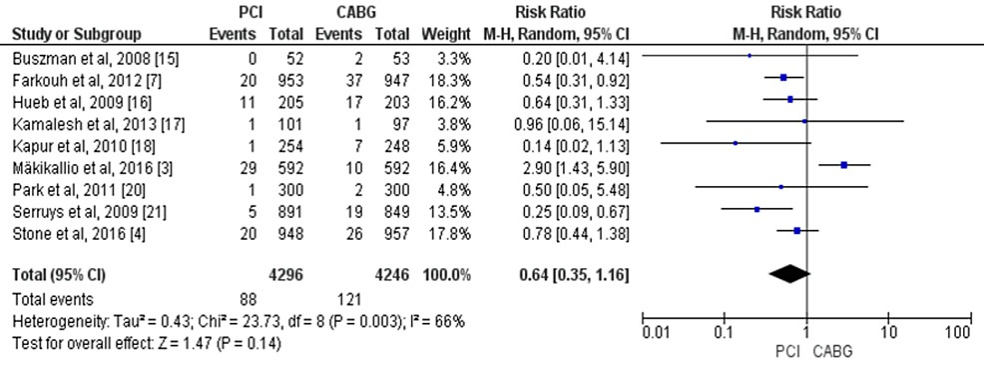
Pooled risk for stroke with PCI versus CABG. Source: References [3,4,7,15-18,20,21]. CABG: coronary artery bypass graft; PCI: percutaneous coronary intervention; 95% CI: 95% confidence interval

Overall, nine studies compared the risk of stroke in patients between two study groups [3,4,7,15-18,20,21]. There was a trend of excess strokes with CABG compared to PCI, but this difference was not statistically significant (RR = 0.64, 95% CI = 0.35-1.16, I^2^ = 66%), as shown in Figure 6.

**Figure 7:**
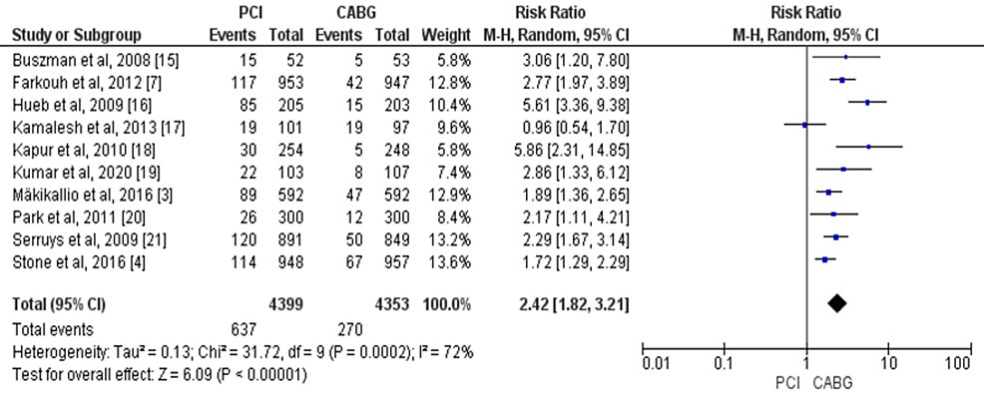
Pooled risk for revascularization with PCI versus CABG. Source: References [3,4,7,15-21]. CABG: coronary artery bypass graft; PCI: percutaneous coronary intervention; 95% CI: 95% confidence interval

Overall, 10 studies compared the risk of revascularization in patients treated with PCI and those treated with CABG enrolling a total of 8,752 patients with CAD [3,4,7,15-21]. The risk of revascularization was significantly higher in the PCI group compared with CABG (RR = 2.42, 95% CI = 1.82-3.21, I^2^ = 72%), as shown in Figure 7.

Subgroup Analysis

Regarding all-cause mortality, a statistically significant difference was observed across multiple subgroups (Table 3). In the subgroup of BMS or early-generation DES (four studies, RR = 1.29, 95% CI = 1.07-1.56) and studies with multivessel CAD (four studies, RR = 1.28, 95% CI = 1.06-1.54). No significant interaction was noted in these stratified analyses as the p-value was more than 0.05.

**Table 3:** Results of the subgroup analysis. *: Significant at p-values <0.05. DES: drug-eluting stents; BMS: bare-metal stents; CAD: coronary artery disease; RR: risk ratio; CI: confidence interval

Outcomes	Subgroups	Number of studies	Total patients	RR (95% CI)	I^2^
All-cause mortality	BMS or early-generation DES	3	2,748	1.29 (1.07-1.56)*	0%
	DES	4	5,190	1.02 (0.78-1.33)	0%
	Left main CAD	5	3,995	1.13 (0.88-1.46)	26%
	Multivessel CAD	4	3,506	1.28 (1.06-1.54)*	20%
Cardiovascular mortality	BMS or early-generation DES	3	2,748	0.84 (0.41-1.72)	72%
	DES	3	4,989	1.26 (0.98-1.63)	0%
	Left main CAD	3	3,689	0.96 (0.59-1.55)	33%
	Multivessel CAD	3	3,296	1.06 (0.67-1.68)	70%
Revascularization	BMS or early-generation DES	3	2,748	3.03 (1.65-5.54)*	52%
	DES	2	3,089	1.79 (1.44-2.23)*	0%
	Left main CAD	4	3,794	1.87 (1.53-2.29)*	0%
	Multivessel CAD	2	618	4.26 (2.21-8.18)*	42%

Regarding cardiovascular mortality, consistent findings were observed in the subgroup of second-generation DES (three studies, RR = 1.26, 95% CI = 0.98-1.63) and BMS or early-generation DES (three studies, RR = 0.84, 95% CI = 0.41-1.72) and in studies with left main CAD (three studies, RR = 0.96, 95% CI = 0.59-1.55) and multivessel CAD (three studies, RR = 1.06, 95% CI = 0.67-1.68).

Discussion

This meta-analysis of 12 RCTs compared long-term outcomes of PCI and CABG for the management of CAD. Based on pooled data from 12 RCTs that included a total of 9,941 patients, of whom 4,954 were assigned to CABG and 4,987 assigned to PCI, we found that PCI was associated with an increased risk of all-cause mortality and repeat revascularization compared to CABG. However, the overall risk of cardiac death, stroke, and myocardial infarction was similar between PCI and CABG. Stratified analysis showed that increased risk for all-cause mortality associated with PCI was only evident in patients with BMS and early-generation DES and multivessel CAD.

Unlike previous systematic reviews and meta-analyses that focused on PCI and CABG in patients either with left main CAD or multivessel CAD [22,23], this meta-analysis aimed to analyze the complete spectrum of unstable and stable syndromes across a range of patient subgroups.

With the advancement of the PCI, such as the design of stents, higher-risk patients with more complex coronary lesions have been included in trials [24]. As such, we found relative mortality benefits of CABG over PCI in this study, especially in patients with multivessel CAD. The findings of this meta-analysis are in line with short-term outcomes reported by Head et al. [25] and other retrospective studies [26,27]. Zhang et al. conducted a meta-analysis [19] and reported no difference in all-cause mortality between PCI and CABG among patients with left main CAD. Subgroup analysis in this meta-analysis identified a similar trend. However, our findings showed that the risk of all-cause mortality is higher in patients with multivessel CAD, and similar findings have been reported in a previous meta-analysis that included only patients with multivessel CAD [23]. Recent propensity-matched research of over 100,000 patients validated the robustness of our results, reporting better survival rates with multivessel CABG compared to multivessel PCI [28]. Current guidelines from the European Society of Cardiology (ESC) and American College of Cardiology (ACC) have recommended that low-complexity multivessel disease can be treated with PCI-like lesions without total occlusions or side branch involvement. On the other hand, more complex multivessel disease (triple-vessel disease) is best managed with CABG [29,30].

A previous meta-analysis conducted among patients with left main CAD showed that the overall risk of stroke was significantly lower in the PCI arm compared to CABG am [31,32]. However, the current meta-analysis showed no significant difference in terms of risk of stroke between CABG and PCI. An in-depth analysis of the Syntax trial [21] and NOBLE trial [3] challenged the benefit of PCI over CABG in the risk of stroke by demonstrating that PCI was associated with the enhanced late stroke that might counteract the early benefit of PCI [3].

One of the benefits of CABG over PCI found in this meta-analysis and in previous meta-analyses [22,23] is the decreased rate of repeat revascularization in the group. Our study’s finding that the PCI group had an increased risk of revascularization than the CABG group is consistent with recent literature [31]. According to observational data, graft patency after CABG is good over the long term, with up to 95% patency in the left internal mammary artery after 15 years [33] and 86% patency in saphenous vein grafts after 10 years [34].

The profound significance of the heart team remains crucial in choosing the best strategy of revascularization for patients with multivessel disease. Current evidence from clinical trials suggests that CABG is preferred to PCI in patients with multivessel disease. The findings of this meta-analysis also support the favorable revascularization of CABG over PCI in patients with multivessel disease.

Limitations

The results of our meta-analysis should be interpreted in light of certain limitations. This was a trial-level meta-analysis as we did not have access to individual patient-level data. Thus, we were not able to perform subgroup analysis to determine whether CABG is superior to PCI for a reduction in all-cause mortality. Moreover, it was limited to certain subgroups of patients such as patients with high syntax scores. Heterogeneity was evident in the analysis of certain outcomes. To incorporate heterogeneity among studies, we used random-effect models for the analysis of those outcomes. We also performed a subgroup analysis to explore the heterogeneity.

## Conclusions

In the pooled data of 9,941 patients with CAD (4,954 in the CABG arm and 4,987 in the PCI arm), CABG was associated with a significant reduction in all-cause mortality and repeat revascularization compared to PCI. This mortality benefit was observed particularly among patients with multivessel CAD. However, no significant difference was reported in the risk of cardiovascular mortality, myocardial infarction, and stroke between the two groups. Considering the risk of revascularization in patients with CAD, CABG needs to be the preferred method of revascularization for patients with CAD. Compared with CABG, PCI with second-generation DES might be a safe strategy for repeat revascularization in patients with CAD; however, it is associated with increased chances of revascularization.
